# Research on Damage Characteristics of Ultrasonic Vibration-Assisted Grinding of a C/SIC Composite Material

**DOI:** 10.3390/s23010224

**Published:** 2022-12-26

**Authors:** Dongpo Wang, Qiushi Liang, Dong Xu

**Affiliations:** 1 School of Mechanical Engineering and Automation, Beihang University, Beijing 100191, China; 2 School of Automation Science and Electrical Engineering, Beihang University, Beijing 100191, China

**Keywords:** C/SiC composite, ultrasonic vibration, grinding force

## Abstract

C/SiC composites are the preferred materials for high temperature resistant (usually above 1500 °C) structural parts in aerospace, aviation, shipbuilding, and other industries. When this kind of material component is processed efficiently by grinding, the damage forms of fiber step brittle fracture and fiber pulling out are often produced on the machined surface/subsurface. The existence of these damage forms deteriorates the quality of the machine surface and may reduce the bending strength of materials to a certain extent. Therefore, it is very important to study the mechanism and the damage law of ordinary grinding and ultrasonic vibration-assisted grinding and take reasonable measures to restrain the machining damage. In this paper, the typical damage forms of C/SiC composites during the end and side grinding are explored. The surface and subsurface damage degree of C/SiC composites during grinding and ultrasonic vibration-assisted grinding were compared. The effects of different process parameters on material damage were compared and analyzed. The results show that the damage forms of ordinary grinding and ultrasonic grinding are basically the same. Compared with ordinary grinding, ultrasonic-assisted grinding can reduce surface damage to a certain extent and subsurface damage significantly.

## 1. Introduction

Since the beginning of the 21st century, with the rapid development of aviation, aerospace, electronics, shipbuilding, automobile, energy, medical, and other fields, new materials, especially new high-temperature structural materials, have become the basis and bottleneck of the research and development of many high-performance hot-end components and even major key equipment. Various fields are increasingly looking forward to the emergence of advanced materials with low density, high strength, high toughness, high modulus, corrosion resistance and high temperature resistance [1]. Ceramic matrix composites have good thermal and mechanical properties, high temperature oxidation resistance, thermal shock resistance and wear resistance, especially fiber-reinforced ceramic matrix composites, which have become a research hotspot at home and abroad [1,2,3,4].

As an important index to evaluate the quality of the workpiece after grinding, surface quality will affect the assembly accuracy, fatigue strength, contact stiffness, and service life of the workpiece during subsequent use. Due to the unique microstructure of carbon fiber reinforced ceramic matrix composites, domestic and foreign scholars have conducted certain research on the damage and surface roughness of conventional grinding and ultrasonic vibration-assisted grinding [5].

Luna G et al. [6] carried out a single abrasive cutting test on SiC_f_/SiC composites and found that when the cutting direction was perpendicular to the fiber direction, although the cutting Angle was 90°, the fiber stripping caused obvious tear damage due to the weak constraint state of the surface fiber material. The damage range of the cutting direction parallel to the fiber direction is basically within the coverage range of abrasive particles. Wang Ben et al. [7] found that the difference between fiber-reinforced resin matrix composites and ceramic matrix composites lies in temperature sensitivity. Due to the poor heat resistance of resin, generally, when the temperature exceeds 200 °C, the resin will soften and the binding ability of the fiber will be sharply reduced. Serious tearing occurseasily during the grinding process. For fiber-reinforced ceramic matrix composites, the ceramic matrix has strong heat resistance, and the composites as a whole show hard and brittle characteristics, with little such tearing damage. Chen J et al. [8] conducted a nanoscything test on C/SiC composites under varying loads, and the results showed that when the loading pole was small, ductility removal behavior existed in both matrix and reinforcement. However, as the load gradually increased, the fiber and matrix would not only fracture but also generate subsurface cracks inside the fiber. Ding Kai et al. [9] used resin bonded diamond wheel to conduct plane grinding experiments on 2D orthogonal braided C/SiC composites and studied the surface/subsurface damage of C/SiC composites by analyzing and measuring the surface morphology, and roughness of carbon fiber region on the grinding surface and grinding subsurface morphology. The results show that the damage forms of carbon fiber are mainly stepped brittle fracture, the subsurface damage forms of carbon fiber are mainly stepped brittle fracture, the subsurface damage forms of SiC are mainly brittle fracture and microcrack, and the damage degree has no obvious difference within the range of experimental parameters.

Yan YY et al. [10] studied the surface or subsurface damage after machining of ZTA ceramic, a high-performance structural ceramic, and its influence on the reliability and life of ceramic parts. The experimental results show that the two-dimensional ultrasonic vibration-assisted grinding technology can remove most of the material in plastic state, reduce the possibility of crack formation and propagation, and significantly reduce the surface damage of ZTA ceramics.

Azrhoushang B et al. [11] conducted finite element modeling and experimental analysis and found that ultrasonic vibration-assisted grinding of C/SiC composites can effectively reduce the surface roughness. Tashiro et al. [12] studied the surface topography of C/SiC composites under conventional grinding and ultrasound-assisted grinding and measured the surface roughness value. They found that compared with ordinary grinding, the surface quality of the workpiece processed by ultrasound-assisted grinding was better, and the measured surface roughness value was also significantly reduced. Khoran et al. [13] conducted a comparison test between ultrasonic assisted grinding and ordinary grinding on C/SiC composites, and the results showed that compared with ordinary grinding, ultrasonic assisted grinding can reduce the surface roughness by 30%. FU et al. [14] respectively conducted ultrasound-assisted grinding and ordinary grinding experiments on C/SiC composites and found that the fiber fracture size after ultrasound-assisted grinding and ordinary grinding was approximately equal, but due to ultrasonic vibration reciprocating hammer machining surface, a large area of fracture occurred in carbon fiber. In addition, it is found that under the condition of ultrasonic vibration assisted grinding, the influence of grinding speed and feed speed on surface roughness does not show a stable law, and Ra increases with the grinding depth increasing.

The aforementioned studies considered the damage generation law and formation mechanism of ultrasonic vibration-assisted grinding. Material properties significantly impact the effect of ultrasonic vibration-assisted grinding, especially for composites with more complex structures. However, studies on the damage generation law and formation mechanism of ultrasonic vibration-assisted grinding of C/SiC composites are limited.

In this experimental study, the damage characteristics of a C/SiC composite subjected to ultrasonic vibration-assisted grinding were evaluated, the C/SiC composite was processed by common grinding and ultrasonic vibration-assisted slope cutting to determine the surface and subsurface defects in side face grinding and end face grinding, the damage formation mechanism was analyzed, and the formation law is summarized to determine the characteristics of ultrasonic vibration-assisted grinding of the C/SIC composite and optimize the material processing theory and processing method.

## 2. Materials and Methods

### 2.1. Experimental Materials

The material used in this experiment was a three-dimensional acupuncture C/SIC composite. A single-layer 0° nonweft fabric, reinforcing mesh, 90° nonweft fabric, and reinforcing mesh were sequentially superimposed to a certain thickness, the fibers in the mesh were vertically pierced into the nonweft fabric by relaying acupuncture to integrate them and to improve the interlayer strength of the C/SIC composite, and the fiber volume fraction of the three-dimensional acupuncture carbon layer prefabricated reinforcement was approximately 40%.

**Figure 1:**
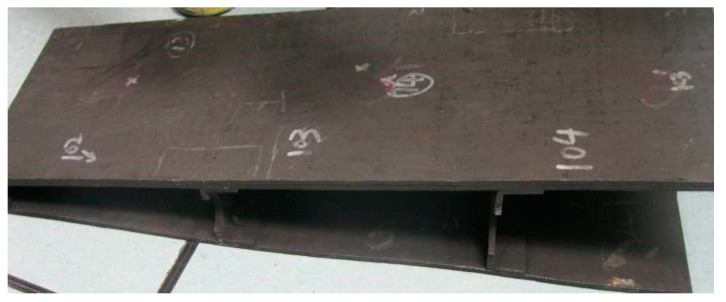
C/SiC composite component.

**Figure 2:**
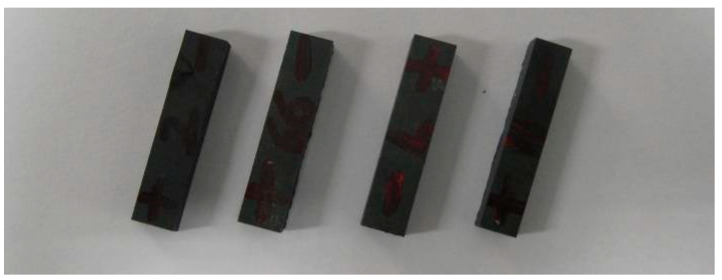
Segmented C/SiC composite specimen.

During the experiment, the C/SIC composite (as shown in Figure 1) was cut into 48 specimens with dimensions of 35 × 8 × 5 mm by an STX-202 diamond wire-cutting machine. Then, 24 specimens were subjected to common grinding, and the other 24 were subjected to ultrasonic vibration-assisted grinding (Figure 2).

### 2.2. Experimental Equipment and Process Parameters

**Figure 3:**
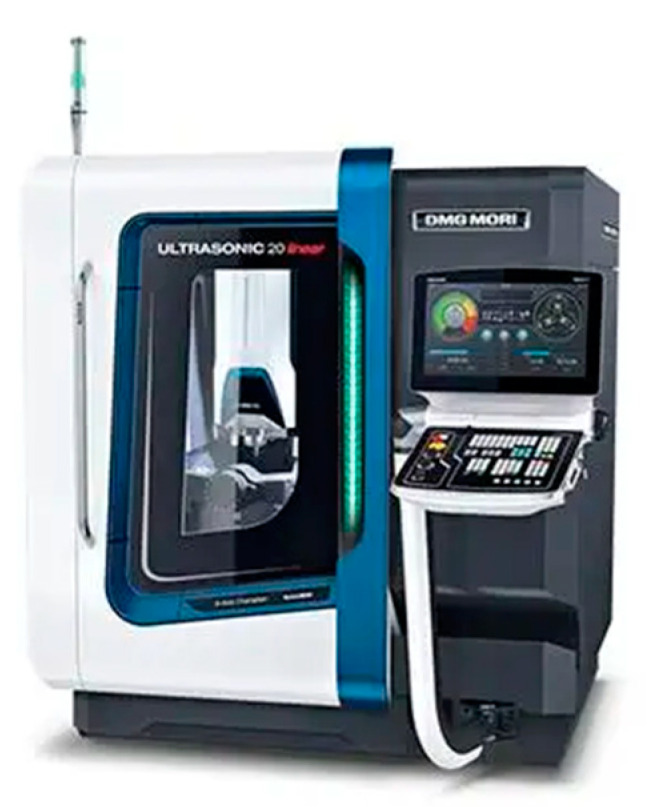
DMG Ultrasonic 20 linear high-speed machining center.

**Figure 4:**
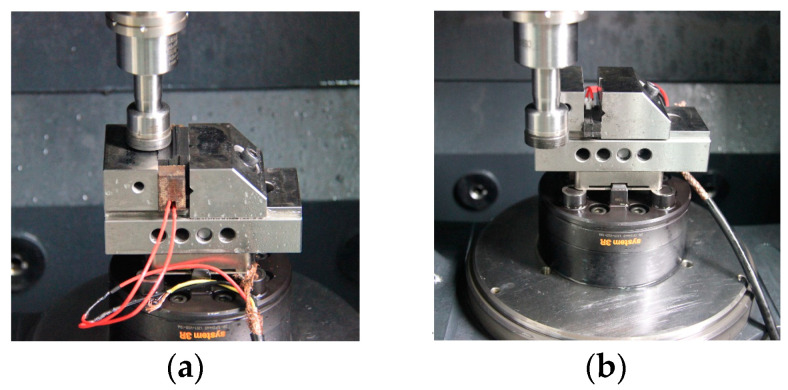
Schematic diagram of ultrasonic vibration-assisted end and side grinding. (**a**) End face grinding. (**b**) Side face grinding.

**Figure 5:**
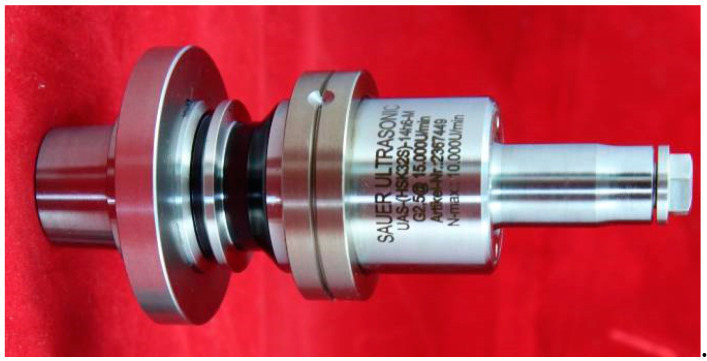
14h6 ultrasonic vibrating shank.

**Figure 6:**
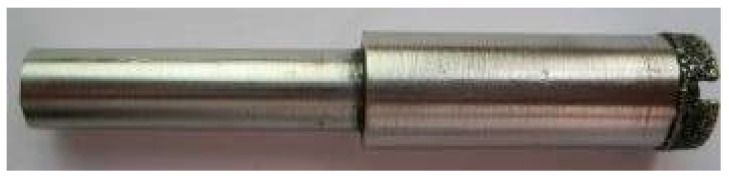
Cup-shaped sintered diamond grinding wheel.

The experiment was performed with a DMG Ultrasonic 20 Linear high-speed machining center. The experimental equipment is shown in Figure 3 The grinding process of end and side face assisted by ultrasonic vibration is shown in Figure 4. Both common grinding (CG) and ultrasonic vibration-assisted grinding (UAG) were performed with a 14h6 ultrasonic tool holder, as shown in Figure 5. A cup-shaped sintered diamond grinding wheel with a diameter of Ø24 mm, a wall thickness of 2 mm, and an average diamond grain diameter of approximately 91 μm was used, as shown in Figure 6. The coolant was a water-based emulsion (Castrol) with a concentration of approximately 4–5%. During the experiment, both internal cooling and external cooling were used, and the internal /external cooling pressure was 20/4 bar.

The specific grinding parameters are shown in Table 1. The side face grinding width b was 5 mm, and the end face grinding width b was 8 mm.

In terms of the selection of ultrasonic vibration characteristic parameters, Dai Bin [15] et al. showed that ultrasonic vibration-assisted grinding of C/SiC composites could change the fracture mode and removal form of fibers under different cutting angles. Compared with the fiber fracture in conventional grinding, the fiber fracture and matrix fracture in grinding process are removed after ultrasonic vibration is applied, and the surface quality is improved. With the increase of ultrasonic amplitude, the fiber damage decreased, the material removal was mainly based on the matrix crushing removal, the material removal rate increased, and the workpiece surface tended to be flat. Based on this factor, the relatively large ultrasonic vibration amplitude A ≈ 6 µm was selected. In order to highlight the ultrasonic high-frequency characteristics, the ultrasonic vibration frequency is relatively large, that is, f = 33.9 KHz.

A Hitachi S-3400N II scanning electron microscope was used to observe the surface and subsurface micromorphology of workpiece grinding and ultrasonic vibration-assisted grinding. Before observation, the specimen surfaces were sprayed with metal.

## 3. Results

### 3.1. Damage Types of C/SiC Composite Grinding

**Figure 7:**
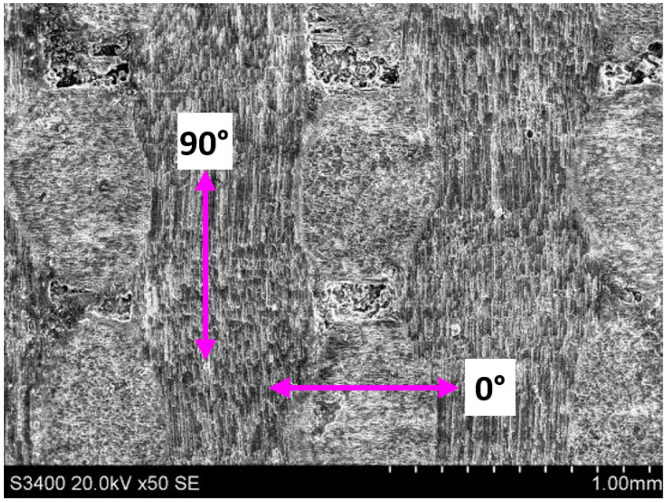
Typical C/SiC grinding surface topography.

Figure 7 shows the surface microstructure of C/SiC composites after grinding. The figure demonstrates that the state of the inter-perpendicular carbon fibers (the 0° fiber is defined as the fiber parallel to the feeding direction, and the 90° fiber is defined as the fiber perpendicular to the feeding direction) after grinding and the pores caused by the shedding of SiC matrix determine the quality of the grinding surface of the C/SiC material.

#### 3.1.1. Damage Types of the C/SiC Composite after Surface Grinding

The surface quality of C/SiC composites after grinding is also mainly determined by the damage of carbon fiber and SiC region. The main forms of damage under this condition are brittle fracture of carbon fiber and brittle spalling of SiC. The fibers at 90° usually appear layered brittle breaking, and the fibers may pull out when the feeding speed or cutting depth is larger. Fiber pull-out and step brittle break are common in 0° fibers. In addition, the pores between the two angles of braided fibers are prone to fracture and collapse defects. In the SiC region, there is a SiC breaking defect.

**Figure 8:**
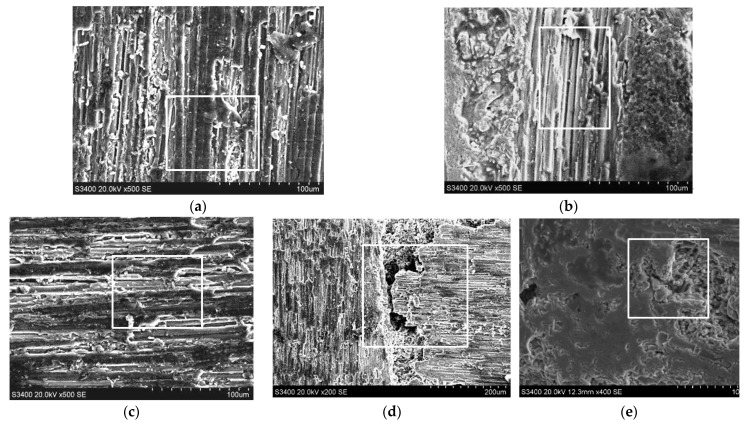
Surface damage types of the C/SiC composite after end face grinding. (**a**) 90° fiber laminar brittle fracture. (**b**) 0° fiber pullout. (**c**) 0° fiber step brittle fracture. (**d**) Pores. (**e**) Collapse fracture in SiC region.

Figure 8 shows the end face grinding microstructure of typical C/SiC composites. In the area shown in the frame in Figure 8a, the surface carbon fiber is removed relatively smoothly, leaving a relatively complete machined surface. However, the underlying carbon fiber was removed as a whole in a large area, showing a multilayer fracture. This is because C/SiC composite material is made of highly brittle silicon carbide matrix and relatively low brittleness carbon fiber composite, the combined strength of the two is not high, when the cutting stress is too large, due to the degree of separation is different, resulting in stratification phenomenon. In the area shown in the frame in Figure 8b, multiple carbon fibers were pulled out, leaving deep gully defects. This is because in the grinding process, the carbon fiber in C/SiC composites will be subjected to bending, stretching, shearing, friction, and other combined effects, when the tensile stress of the fiber is greater than its own tensile strength, the fiber will be pulled off, the formation of fiber breakage defect. When the bending stress of the fiber is greater than the torsional strength of the fiber itself, the strength of the matrix is higher than that of the carbon fiber, so part of the fiber will be torn from the parent or the whole fiber will be pulled out, forming a gully defect. In the area shown in the frame of Figure 8e, the fracture surface is multi-tiered, and the fracture is disorderly, with large epitaxial damage. In the area shown in the frame in Figure 8d, it can be seen that the fiber direction is 0° extending left and right, while the fiber direction on the left side of the frame is 90° extending up and down. Deep pore defects appeared in the frame area at the junction of 0° and 90° fibers, which were mainly caused by the upper 0° fiber being worn off and the left fiber being broken. At this time, when the abrasive tip contacted the carbon fiber here, due to the weak strength of the joint, the tooltip pulled out the matrix material with relatively low connection strength at the pore and some vertical needled fibers, resulting in local pores. In the area shown in Figure 8e, relatively serious cracking defects in SiC region appear, which are mainly caused by the peeling effect of grinding edge, and the fracture form is a typical brittle fracture.

**Figure 9:**
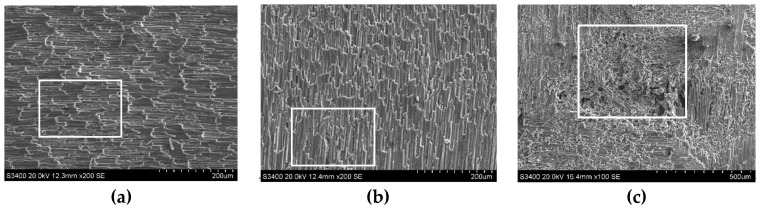
Surface damage types of the C/SiC composite after side face grinding. (**a**) 90° fiber step brittle fracture. (**b**) 0° fiber step brittle fracture. (**c**) Pores.

The typical side face grinding microstructure of C/SiC composites is shown in Figure 9. As can be seen from the figure, surface material defects of C/SiC composite side face grinding mainly include brittle fracture of fiber bundles around pores (at braided angles), stepped brittle fracture of carbon fibers in the region, and the fracture morphology is an irregular curve. Figure 9a Within the entire horizon, fiber fractures show obvious stepped brittle fracture characteristics, with larger layered ripples, more layers, and more irregular cross sections. In addition, there are more fiber fractures and pull-out defects in the area shown in the frame. Figure 9b shows the characteristics of stepped brittle fracture similar to that in Figure 9a, but the corrugations are obviously dense. The area shown in the frame is the intersection of 0° carbon fiber on the left and 90° carbon fiber on the right, and the SiC area below the frame. The fibers can be seen flaking at the intersections, affecting the fiber regions and SiC regions on both sides, resulting in the extension of the defect.

#### 3.1.2. Subsurface Grinding Damage Types of the C/SiC Composite

##### End Face Grinding

**Figure 10:**
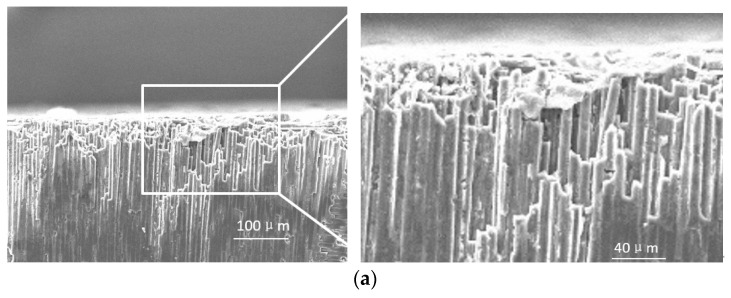

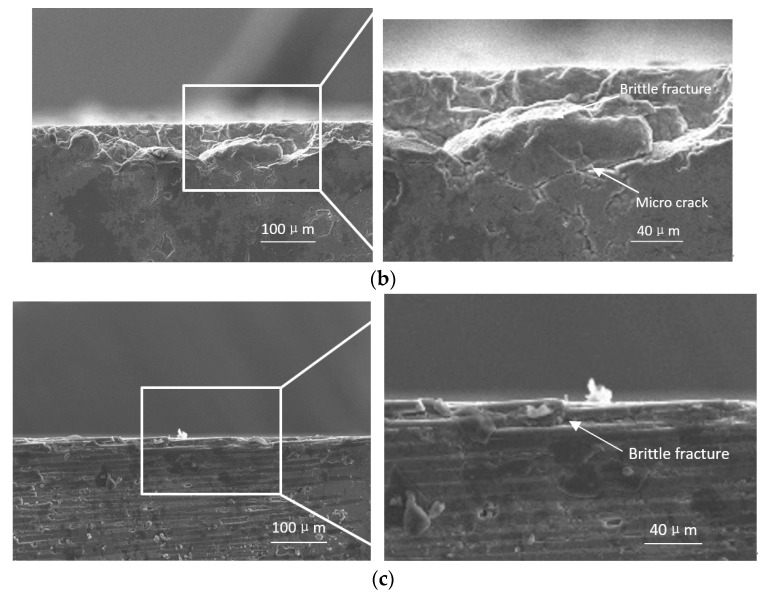
Subsurface damage types of the C/SiC composite after end face grinding. (**a**) 90° carbon fiber step brittle fracture. (**b**) The SiC matrix is a brittle fracture and spalling. (**c**) 0° carbon fiber edge brittle fracture.

The main damage forms on the subsurface of C/SiC composites are stepped brittle fracture of carbon fibers at the direction of 90°, and brittle fracture and spalling of SiC matrix between carbon fibers occur when the feed rate or cutting depth is larger. The damage of 0° carbon fiber is very small and rarely appears in the edge region of the sample. As shown in Figure 10a, the brittle fracture of carbon fibers at the 90° subsurface direction is relatively serious, showing irregular fracture, and complex fault, accompanied by fiber fracture and fiber pulling out. As shown in Figure 10b, the SiC region on the subsurface mainly presents brittle fracture and breakage defects, and obvious microcracks can be seen. The brittle fracture and breakage are mainly caused by the impact of the grinding edge and the knock-out effect during SiC grinding. Part of the microcracks is in the formation process of C/SiC composites. Due to the poor density of the materials, there are already micro cracks on the surface, which are only exposed after grinding. More because the silicon carbide matrix is brittle, when the grinding force is large, the irregular brittle section will lead to the microcracks will lead to the matrix fracture and the formation of microcracks. As shown in Figure 10c, relatively clear fiber brittle breakage occurs at the edge of 0° fiber, which is mainly caused by a large impact at the edge.

##### Side Face Grinding

**Figure 11:**
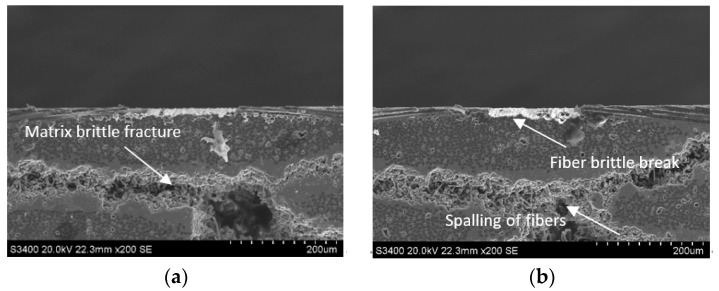
Brittle fracture and peeling damage of the carbon fibers on the subsurface of the C/SiC composite after side face grinding. (**a**) SiC matrix area defects. (**b**) Carbon fiber defect.

The main subsurface damage of side face grinding is a brittle fracture of the matrix, crack propagation, brittle fracture and spalling of carbon fiber, etc. As shown in Figure 11a, under the influence of interaction between abrasive particles and matrix materials, impact force, and transverse and longitudinal crack propagation caused by surface defects such as carbon fiber pulling out, the SiC region on the subsurface presents the characteristics of brittle fracture with very irregular section. As the crack grows and the material is taken out by the abrasive particles, the brittle fracture characteristics are banded. As can be seen in Figure 11b, brittle fracture occurred on the carbon fiber at the edge of the material, which was mainly due to the fact that the carbon fiber in the subsurface layer was subjected to shear and extrusion at the same time and was in a state of stress concentration under the action of abrasive cutting. When the internal stress of the material exceeds the tensile strength of the material, the fiber fracture occurs at the stress concentration position. While the fiber spalling in the middle is mainly caused by the fact that the shear resistance of carbon fiber is weaker than that of SiC matrix during the normal grinding along the fiber. During the grinding of C/SiC composite material, the carbon fiber is taken out by the cutting edge and separated from the matrix, and the holes caused by fiber spalling appear on the machining surface.

### 3.2. Impact of Ultrasonic Vibration on the Grinding Surface/Subsurface Damage of C/SiC Composite

#### 3.2.1. Impact of Ultrasonic Vibration and Process Parameters on the Surface Damage of the C/SiC Composite Material after Grinding

##### End Face Grinding

**Figure 12:**
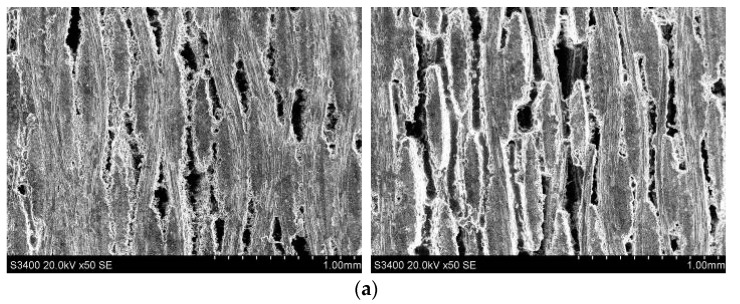

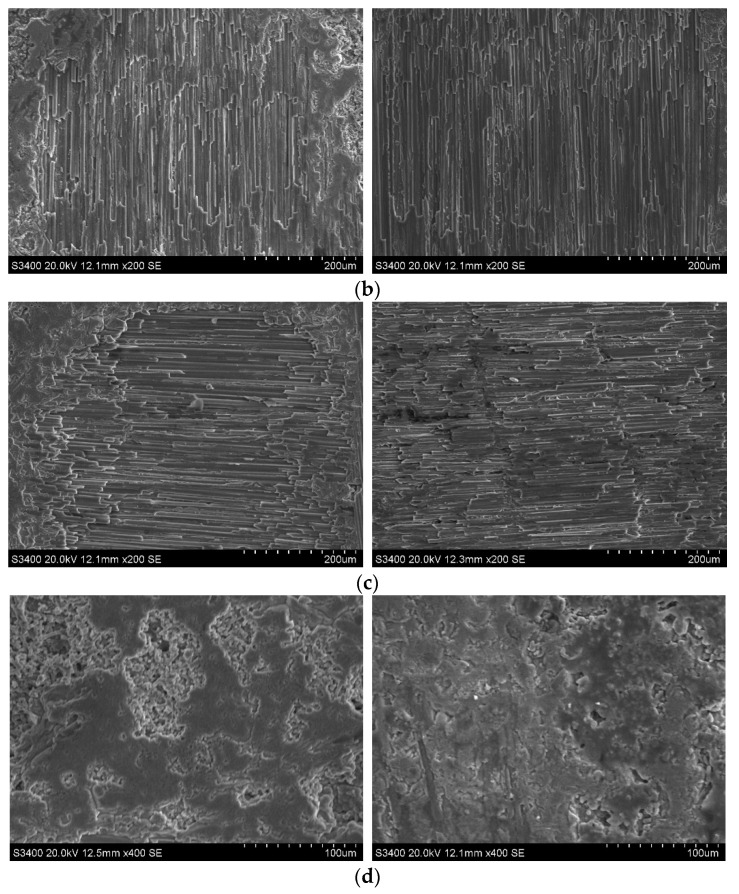
Micromorphology of the C/SiC composite after end face grinding: (left) CG; (right) UAG(v_s_ = 12.6 m/s, a_p_ = 0.1 mm, v_w_ = 0.05 m/min). (**a**) Pores. (**b**) 0° fibers. (**c**) 90° fibers. (**d**) SiC region.

Figure 12 shows the end face micromorphology of the C/SiC composite after typical common grinding and ultrasonic vibration-assisted grinding (v_s_ = 12.6 m/s, a_p_ = 0.1 mm, v_w_ = 0.05 m/min). The figure demonstrates that the pore morphology and SiC region morphology after common grinding and ultrasonic vibration-assisted grinding are not substantially different, but the fiber fracture trend diminishes. As can be seen from Figure 12b, during 0° grinding, the overall grinding surface of CG machining presents layered fractures with multiple layers, irregular machining edges, and large damage, and material breaking defects appear in the picture frame. However, UAG processing has less stratification, a more regular edge, and less local damage. As can be seen from Figure 12c, during 90° grinding, the morphology of CG machining and UAG machining is significantly different. The fiber fracture layers of the CG machining surface frame are significantly more than that of UAG machining, showing a multi-layered ladder shape with 6–7 layers. However, UAG processing presents a smooth overall shape, less stratification, and less edge damage. As can be seen from Figure 12d, the SiC region processed by CG is obviously characterized by large size, deep depth, and visible cracks on the surface, and the machining damage is more obvious than that in UAG processing.

##### Side Face Grinding

**Figure 13:**
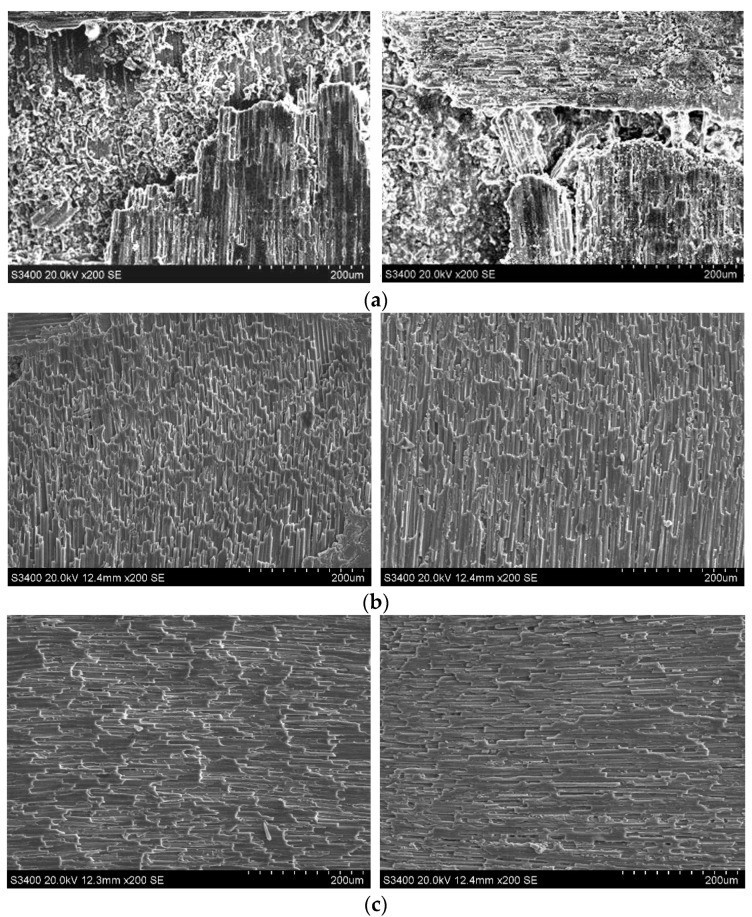
Surface micromorphology of the C/SiC composite material after side face grinding: (left) CG; (right) UAG(v_s_ = 12.6 m/s, v_w_ = 100 mm/min, a_p_ = 0.1 mm). (**a**) Pore micromorphology. (**b**) 0° fibers. (**c**) 90° fibers.

Figure 13 shows the typical surface micromorphology of the side face of the C/SiC composite after common grinding and ultrasonic vibration-assisted grinding. As can be seen from Figure 13a, there is little difference in pore size and shape between the two. As can be seen from Figure 13b, the CG machining surface is more densely fractured and layered, and there are obvious fiber pulling defects in the position shown in the frame. As can be seen from Figure 13c, CG processing presents relatively prominent step fracture and stratification features. According to the comparison between CG and UAG processing under these parameters, the fracture size at the fiber braided Angle, the step fracture size of carbon fiber and the brittle fracture size of SiC in UAG processing are slightly better than those in CG processing, but in general, there is no significant difference between them.

Comparing Figure 12 with Figure 13 shows that with the same processing method and the same parameters, the fiber damage size is smaller than those after end face grinding. This corresponds to the side grinding force being less than the end grinding force.

#### 3.2.2. Impact of Ultrasonic Vibration and Process Parameters on the Subsurface Damage of the C/SiC Composite Material after Grinding

##### End Face Grinding

**Figure 14:**
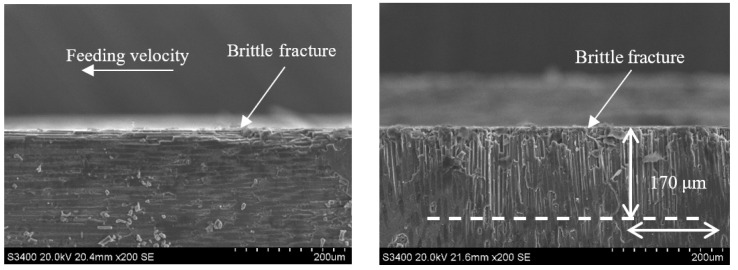
Subsurface topography of the CG-C/SiC composite (v_s_ = 1.26 m/s, a_p_ = 0.1 mm, v_w_ = 100 m/min).

**Figure 15:**
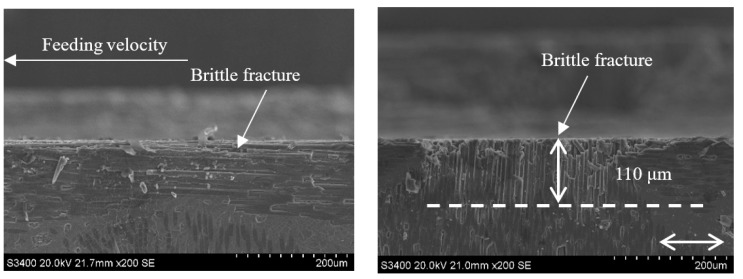
Grinding subsurface topography of the UAG-C/SiC composite (v_s_ = 1.26 m/s, a_p_ = 0.1 mm, v_w_ = 100 m/min).

**Figure 16:**
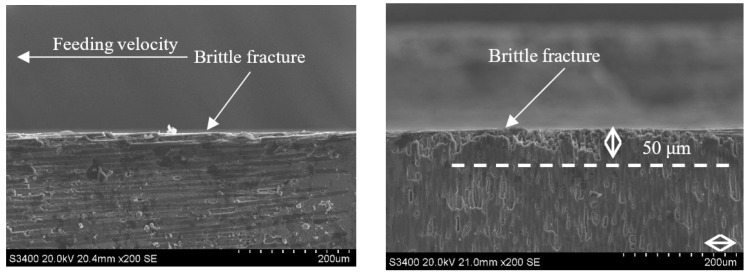
Grinding subsurface topography of the CG-C/SiC composite (v_s_ = 12.6 m/s, a_p_ = 0.1 mm, v_w_ = 100 m/min).

**Figure 17:**
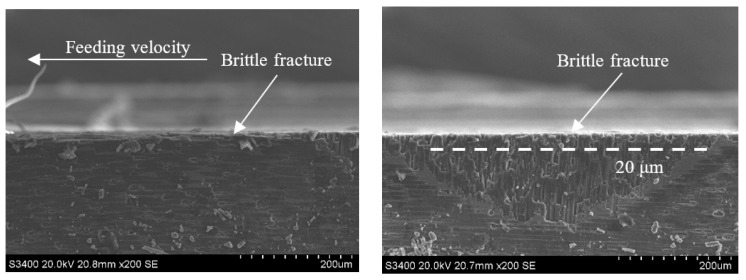
Grinding subsurface topography of the UAG-C/SiC composite (v_s_ = 12.6 m/s, a_p_ = 0.1 mm, v_w_ = 100 m/min).

**Figure 18:**
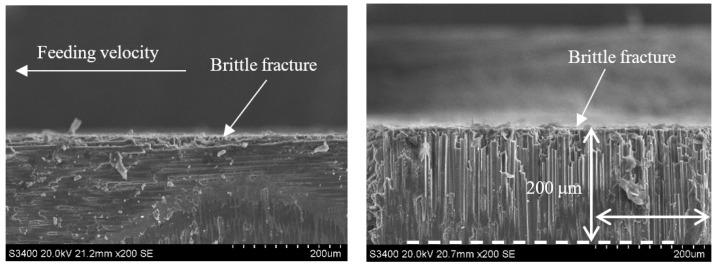
Grinding subsurface topography of the CG-C/SiC composite (v_s_ = 1.26 m/s, a_p_ = 0.4 mm, v_w_ = 100 m/min).

**Figure 19:**
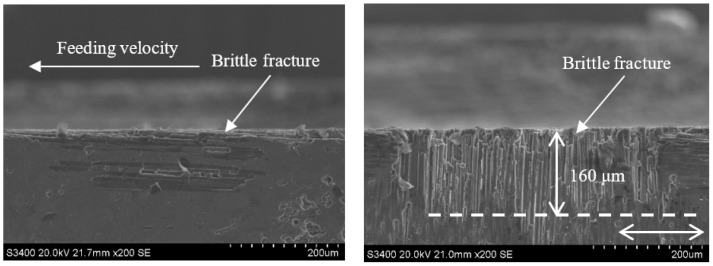
Grinding subsurface topography of the UAG-C/SiC composite (v_s_ = 1.26 m/s, a_p_ = 0.4 mm, v_w_ = 100 m/min).

**Figure 20:**
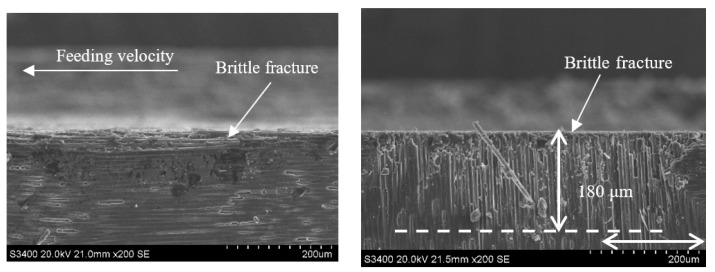
Grinding subsurface topography of the CG-C/SiC composite (v_s_ = 1.26 m/s, a_p_ = 0.1 mm, v_w_ = 500 m/min).

**Figure 21:**
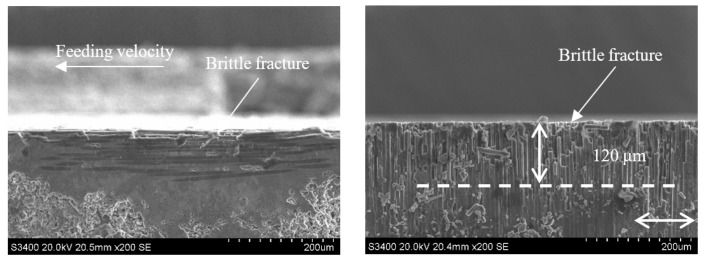
Grinding subsurface topography of the UAG-C/SiC composite (v_s_ = 1.26 m/s, a_p_ = 0.1 mm, v_w_ = 500 m/min).

During end face grinding, the subsurface micromorphologies of typical C/SiC composites obtained by ordinary grinding and ultrasonic assisted grinding are shown in Figure 14, Figure 15, Figure 16, Figure 17, Figure 18, Figure 19, Figure 20 and Figure 21. It can be seen from the figure that the subsurface damage forms of C/SiC composites obtained by grinding are basically the same whether it is ordinary grinding or ultrasonic assisted grinding, which mainly include brittle fracture of carbon fiber, brittle fracture of SiC matrix between carbon fiber and microcracks in SiC matrix. By comparing the subsurface morphology of CG machining and UAG machining under the same conditions, it can be seen that compared with CG machining, UAG machining can significantly reduce the subsurface damage size of carbon fiber perpendicular to the direction of feed velocity, which is different from the surface morphology characteristics of grinding.

Figure 14 and Figure 15 show the subsurface topographies after common grinding and ultrasonic vibration-assisted grinding under the conditions of v_s_ = 1.26 m/s, a_p_ = 0.1 mm, and v_w_ = 100 m/min. After CG and UAG processing, the main damage patterns in the 0° carbon fiber zone are brittle fracture and cracking at the edge. By comparison, it can be seen that the two methods have no obvious influence on the damage size of the 0° carbon fiber bundle. However, in the 90° carbon fiber region, the brittle fracture depth decreases from 170 μm in CG processing to 110 μm in UAG processing. The damage depth, number of brittle faults, and section quality of UAG processing were significantly better than those of CG processing. This is mainly due to the superposition of ultrasonic vibration, which increases the grinding distance, reduces the time of the grinding edge to contact the material, reduces the grinding force, and effectively reduces the damage depth and damage range.

Comparing Figure 14 with Figure 16 and Figure 15 with Figure 17 reveals that under the conditions of a_p_ = 0.1 mm and v_w_ = 100 m/min, when the grinding speed v_s_ increases from 1.26 m/s to 12.6 m/s, the size of the damage in the carbon fiber region perpendicular to the feeding direction generated by each method is significantly reduced from 170 μm and 110 μm to 50 μm and 20 μm, respectively. This is because, when the rotating speed is low, the axial force on the surface layer of the material is relatively large. Due to the difference in the elastic modulus of the carbon fiber and SiC matrix, the deformation coordination ability of the interface between the fiber and the matrix is different, and the fiber detachment, pull out, and matrix cracking is more likely to occur. However, with the increase in rotational speed, the damage of CG machining and UAG machining significantly decreased, and the plastic cutting area increased, indicating that the high rotational speed was conducive to the plastic removal of materials and the reduction of damage. As can be seen from the comparison between Figure 16 and Figure 17, the surface quality of UAG processing is excellent at a high rotation speed and is obviously better than that of CG processing. This is due to the superposition of ultrasonic vibration, which results in faster surface material removal at higher speeds with less cutting force and less depth and scope of the damage. It also shows that UAG is more suitable for high-speed machining, which is very important for improving machining efficiency. 

Comparing Figure 14 with Figure 18 and Figure 15 with Figure 19 demonstrates that under the conditions of v_s_ = 1.26 m/s and v_w_ = 100 m/min, when the grinding depth a_p_ is increased from 0.1 mm to 0.4 mm, the size of the damage in the carbon fiber region perpendicular to the feeding direction generated by each method increases from 170 μm and 110 μm to 200 μm and 160 μm, respectively. This is because, when the grinding depth is small, the disbonding between the fibers and the substrate on the grinding surface of the material is limited to a relatively regular range. The grinding surface of the material is relatively flat, only a few layers of fibers are cut by the abrasive particles, the fracture sweep scope is small, and the fracture is relatively neat. With the increase of grinding depth, there are obvious cracks between the fiber bundles, the larger the crack area and the longer the crack propagation length, the phenomenon of fiber delamination is very obvious. By comparing Figure 18 and Figure 19, it can be seen that the damage of CG is more obvious, and the fibers in the 90° direction show severe delamination and zigzag defects, which are more prominent than that when ap = 0.1 mm. This difference is mainly due to the periodical vibration change of cutting depth caused by ultrasonic vibration, and the impact accelerates the rapid breaking and removal of surface materials. Thus, the subsurface damage does not change dramatically after the cut depth increases obviously, which is also the advantage of ultrasonic vibration grinding. 

Comparing Figure 14 with Figure 20 and Figure 15 with Figure 21 reveals that under the conditions of v_s_ = 1.26 m/s and a_p_ = 0.1 mm when the feeding velocity v_w_ increases from 100 mm/min to 500 mm/min, the size of the damage in the carbon fiber region perpendicular to the feeding direction generation by each method increases from 170 μm and 110 μm to 180 μm and 120 μm, respectively, which is not significant. This is because, when the feed rate of abrasive particles is low, the grinding surface of the material is relatively flat, and the surface fiber is almost not stratified and pulled out, while when the feed rate is high, there are multiple layers of fiber on the surface of the material are affected, the material stratification is obvious, and the width and length of the crack are significantly increased. The interfacial layer of the fiber bundle is cracked, and the fiber and matrix are debonded, and then a large area of crack is formed. By comparing Figure 20 and Figure 21, it can be seen that the depth of damage in UAG machining is significantly less than that in CG machining. This is because the introduction of ultrasonic vibration weakens the trend of increasing the cutting depth of each abrasive particle and the material volume removed by each abrasive particle, thus weakening the cutting impact effect and reducing the damage depth and area during material cutting. In ultrasonic vibration assisted machining, it is beneficial to improve the machining efficiency to increase the feed speed as much as possible without affecting the machining surface quality. 

##### Side Face Grinding

**Figure 22:**
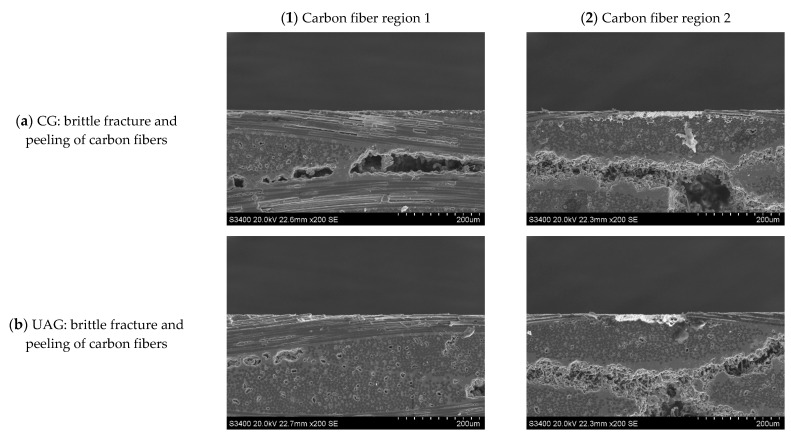
Subsurface morphologies of typical the C/SiC composite material after common grinding and ultrasonic vibration-assisted grinding(v_s_ = 1.26 m/s, v_w_ = 100 mm/min, a_p_ = 0.1 mm).

**Figure 23:**
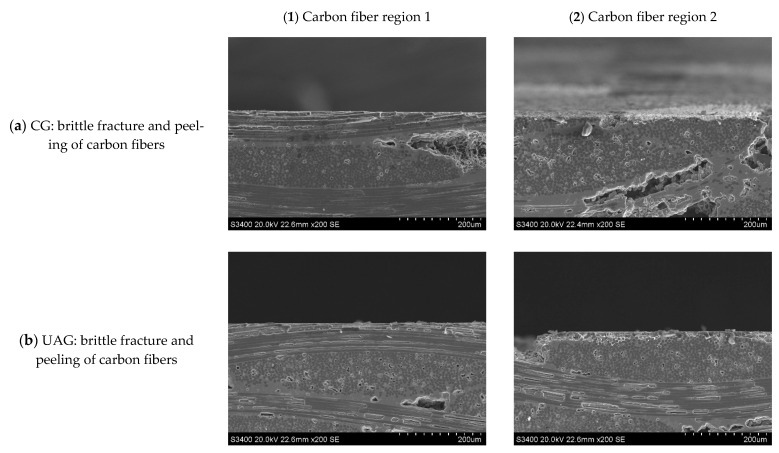
Subsurface morphologies of the C/SiC composite material after common grinding and ultrasonic vibration-assisted grinding(v_s_ = 12.6 m/s, v_w_ = 100 mm/min, a_p_ = 0.1 mm).

**Figure 24:**
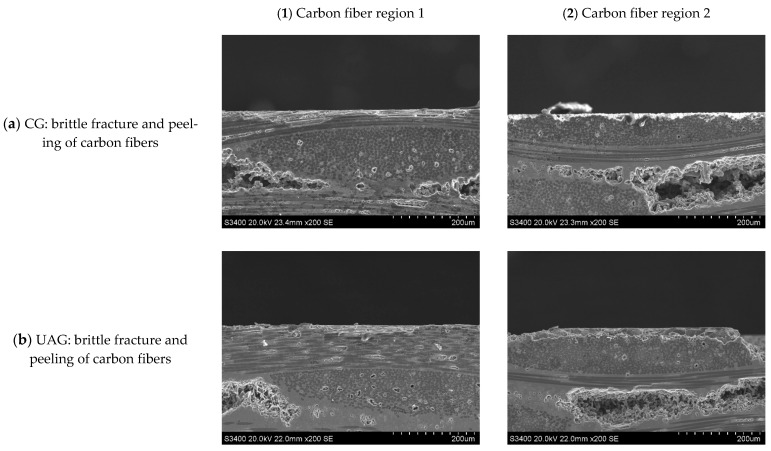
Subsurface morphologies of the C/SiC composite material after common grinding and ultrasonic vibration-assisted grinding(v_s_ = 12.6 m/s, v_w_ = 100 mm/min, a_p_ = 0.2 mm).

**Figure 25:**
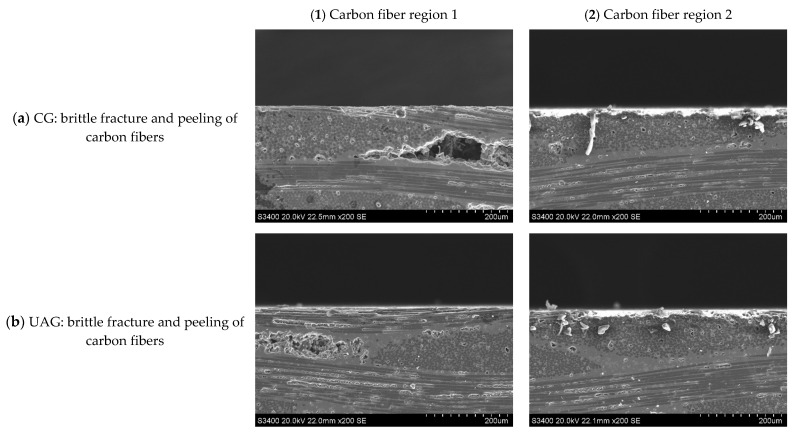
Subsurface morphologies of the C/SiC composite material after common grinding and ultrasonic vibration-assisted grinding(v_s_ = 1.26 m/s, v_w_ = 500 mm/min, a_p_ = 0.1 mm).

Typical subsurface morphologies of the C/SiC composite after grinding are shown in Figure 22, Figure 23, Figure 24 and Figure 25. The figures show that after both common grinding and ultrasonic vibration-assisted grinding, the subsurface damage forms are carbon fiber brittle fracture and peeling. Under the same parameter conditions, the subsurface damage degree of UAG processing is slightly lower than that of CG processing.

Comparing Figure 22 with Figure 23, Figure 24 and Figure 25 reveals a conclusion similar to that reached for end face grinding, which is not repeated here.

## 4. Conclusions

This paper reports a comparative experimental study on a C/SiC composite material after common grinding and ultrasonic vibration-assisted grinding. The law of variation in surface/subsurface damage with different processing methods and process parameters was analyzed. The main conclusions are as follows:

1. The main form of surface damage of the C/SiC composite was carbon fiber brittle fracture. In end-face grinding, the 90° fibers generally experienced a laminar brittle fracture, and some fibers were pulled out under large process parameters. The 0° fibers generally appeared in step brittle fracture. In addition, the pores between the fibers woven in two angles were prone to fiber breakage and collapse. SiC collapse defects were observed in the SiC region. In side-face grinding, the material defects were mainly brittle fractures of the fiber bundles around the pores (woven corners) and step brittle fractures in the carbon fiber region, and the fracture morphology was irregular curves.
2. The main damage forms of the subsurface of the C/SiC composite after grinding were mainly carbon fiber step brittle fracture and SiC matrix brittle fracture and peeling between the carbon fibers.
3. In end- and side-face grinding, ultrasonic vibration-assisted grinding did not significantly reduce the grinding surface damage compared with common grinding under the same conditions, but it did significantly reduce the subsurface damage size of the carbon fibers perpendicular to the feeding direction.
4. In the grinding of the C/SiC composite material, changing the process parameters greatly impacted the 90° fibers but only slightly impacted the 0° fibers.
5. From the perspective of the process parameters, increasing the grinding depth during ultrasonic vibration-assisted grinding increased the subsurface damage size, and increasing the grinding speed decreased the subsurface damage size. The feeding velocity had little impact. Therefore, from the perspective of suppressing machining damage without degrading the machining efficiency, a smaller grinding depth, a higher grinding speed, and a higher feeding velocity should be chosen for C/SiC composite materials in ultrasonic vibration-assisted grinding.

## Figures and Tables

**Table 1:** C/SiC composite grinding parameters.

	Factor	Linear Velocity of Grinding Wheel *v*_s_ (m/s)	Feeding Velocity *v*_w_ (mm/min)	Grinding Depth *a*_p_ (mm)
Test				
1		1.26	50	0.1
2		1.26	100	0.1
3		1.26	500	0.1
4		1.26	100	0.4
5		12.6	50	0.1
6		12.6	100	0.1
7		12.6	100	0.2

## Data Availability

Not applicable.
